# Breaking the one-to-one mapping: a reconfigurable topological haptic proxy for astronaut VR training

**DOI:** 10.1093/nsr/nwag160

**Published:** 2026-03-13

**Authors:** Qinglin Jiang, Yuguang Ma

**Affiliations:** Institute of Polymer Optoelectronic Materials and Devices, Guangdong Basic Research Center of Excellence for Energy and Information Polymer Materials, State Key Laboratory of Luminescent Materials and Devices, South China University of Technology, China; Guangdong Provincial Key Laboratory of Luminescence from Molecular Aggregates, South China University of Technology, China; Institute of Polymer Optoelectronic Materials and Devices, Guangdong Basic Research Center of Excellence for Energy and Information Polymer Materials, State Key Laboratory of Luminescent Materials and Devices, South China University of Technology, China; Guangdong Provincial Key Laboratory of Luminescence from Molecular Aggregates, South China University of Technology, China

Virtual reality (VR) has unlocked unprecedented frontiers for immersive sensory experiences. Central to these advancements are haptic-VR interfaces, often referred to as haptic proxies, which govern the intricate interactions between users and virtual entities while delivering tactile feedback [1].

Serving as a cognitive bridge between virtual and physical domains, these devices establish a correspondence with virtual objects across at least one physical dimension, such as texture or geometry, while enabling real-time mapping of physical manipulations onto their virtual counterparts [2]. Active haptic proxies typically generate electro-tactile feedback through wearable systems or rely on actively deforming props; passive proxies prioritize the intrinsic tactile cues of physical objects. Notably, interactions based on physical contact provide users with direct proprioceptive feedback from their own actions. Consequently, passive haptic proxies (PHPs) exhibit immense potential for enhancing prop-based VR environments, where the sense of realism is paramount [3].

PHPs encounter significant bottlenecks when addressing the demands for large-scale model variety and rapid VR reconstruction. Unlike active devices that can autonomously alter their morphology to suit diverse scenarios, passive counterparts are typically characterized by a fixed geometry [4]. While a static form simplifies the initial mapping of virtual models, the necessity for a unique physical prop for every virtual object inevitably leads to prohibitive costs, storage challenges and material waste, limiting the scalability and sustainability of passive tactile systems.

**Figure 1.**
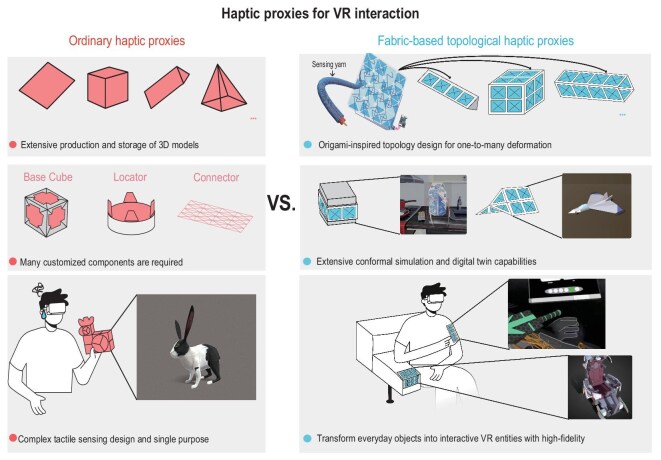
Comparison of ordinary proxies (left) with FTHP (right).

The authors introduce an origami-inspired fabric topological haptic proxy (FTHP) [5]. This system synergistically integrates heterogeneous rigid–flexible segments with dual-level S/Z-twist triboelectric yarns, enabling the fabric to transition between diverse morphologies while maintaining structural stability. By leveraging material-specific triboelectric signatures, the FTHP facilitates the real-time, non-storage-based conversion of physical deformations into interactive virtual entities, effectively eliminating the need for pre-stored 3D replicas. This comprehensive study provides a definitive blueprint for systematic scientific research, spanning from precise material selection and device fabrication to the ultimate validation of VR feasibility, and paves the way for the development of next-generation VR based flexible haptic proxies. (Fig. 1).

At the functional core of the FTHP are 16 topological units engineered with varied configurations of polytetrafluoroethylene (PTFE) and nylon building blocks. This heterogeneous arrangement generates 16 distinct, decoupled electrical signatures, allowing for localized interactions. Facilitated by flexible hinges between these rigid segments, the FTHP theoretically accommodates a vast library of morphological transformations. The origami-inspired architecture provides the necessary structural support to stabilize the output signals and maintain the integrity of the rendered VR model. Physical reconfiguration triggers an instantaneous update of the virtual geometry. This real-time physical-to-virtual synchronization resolves the cumbersome nature of passive proxies, offering a streamlined and adaptable interface for next-generation VR interactions.

Furthermore, the authors delineate an advanced operational framework to evaluate the precision and stability of interactions across three distinct states. A convolutional neural network (CNN) is implemented to enhance recognition efficiency and long-term reliability, achieving a classification accuracy exceeding 92% across all three interaction modalities and 14 distinct actions. This validates its ability to function as a dynamic, multi-purpose tool—from a flat touchpad to various 3D controllers—bypassing the need for conventional one-to-one physical–virtual mapping.

Overall, the authors proposed that engineered topology, rather than complex hardware, can provide the core intelligence for interpreting physical interaction. This FTHP paves a sophisticated new avenue for VR technology, fundamentally addressing the long-standing challenges of high cost and limited reusability in conventional tactile interfaces.
